# An Editorial Introduction to Critical Thinking in Everyday Settings

**DOI:** 10.3390/jintelligence13070084

**Published:** 2025-07-09

**Authors:** Christopher P. Dwyer

**Affiliations:** Technology Education Research Group (TERG), Department of Technology Education, Technological University of the Shannon, Midlands Midwest, N37 HD68 Westmeath, Ireland; christopher.dwyer@tus.ie

When I was first invited to guest edit a Special Issue of the *Journal of Intelligence* on critical thinking (CT), I was quite excited by the prospect for what I saw as two important reasons. First, it would give me the opportunity to collaborate with so many great researchers in the field (including both established CT researchers who I have admired for many years and early career researchers who are doing some really exciting work)—each of whom I want to thank so very much for their great work and contribution to this Special Issue. This collection of work would not be possible without their efforts and insights. Indeed, the field of CT owes them their gratitude! Second, the prospect of editing a Special Issue gave me a chance to sit down and really think about what actually *needs* addressing in the world of CT research, more broadly than one’s own research, and the potential contribution such focus could make.

Critical thinking is a metacognitive process that, through purposeful, self-regulatory reflective judgment; skills of analysis, evaluation, and inference; and a disposition towards thinking, increases the chances of producing a logical conclusion to an argument or a solution to a problem. Notably, as a result of the dramatically rising availability of information (including both misinformation and disinformation), the need for CT is arguably more important now than ever. While application of CT in ‘everyday settings’ might seem as an obvious focus of CT research (especially in light of zeitgeist perceptions at present in our world around us), it may come as surprising to many not entirely familiar with the field that, arguably, it is not really where the research spotlight typically shines. Among the most widespread focuses of CT research is conceptualising and defining it. Indeed, CT research has been commonly doing this since the late 1980s/early 1990s—and that is not a criticism, per se. To a large extent, I still really enjoy reading these conceptual manuscripts on CT, regarding what it is and how we do it. However, there comes a point where you can only read so many papers that say more or less the same thing about CT. Too often, after reading such manuscripts, I am left wondering about the applicability of this in a meaningful, practical way that has not already been explored countless times before. What does this actually mean for the world, the people and the thinking around us—outside the academia bubble?

Likewise, we know CT is important; otherwise, we would not put so much of our time and effort into researching it. But just because it is important does not mean everyone else recognises that, let alone knows how to do it. As a result, another large bulk of the CT research focuses on how to create this capacity and improve it—which, of course, we typically do through teaching and learning. Indeed, making students interested in improving their decision-making and teaching them how to think critically is a noble cause, with CT representing a commonly desired educational outcome by not only educators but employers and society more generally. However, the problem with this focus is that it generally limits discussions of CT to educational settings, which is further problematic because it alienates individuals who did not have or will not have the opportunity to attend third-level education (where CT is most commonly taught in an explicit manner) or, perhaps more broadly, individuals who are simply just not part of the academic world.

Do not get me wrong; understanding what CT refers to and how we can use that conceptualisation to improve it through training are fundamental to the goals of researchers in CT. Indeed, most of my research career has focused on these very things. However, what is equally important is understanding the practicalities surrounding CT’s application in the real world. Moreover, this is not to say that CT research (such as that presented in this Special Issue) should not or no longer discuss CT in conceptual or educational contexts. Rather further efforts need to be made in the research, be it with respect to further integration within methodological rationales or targets for enhancement, to address real-world issues and applications (and not just as a token point in a study’s discussion section), so as to ensure clarity regarding how and why CT is so important for our world’s societies. Furthermore, this is why I was—and am—so excited about this Special Issue on “Critical Thinking in Everyday Settings”. It represents a collection of some really interesting pieces that encourages its authors and readers alike to step out into the real world, where CT can be discussed in terms of wide-ranging applications.

Given the role of higher-order cognitive processes at the core of CT, the relationship between intelligence and CT is important for consideration not only for readers of the *Journal of Intelligence* (within which this Special Issue on CT calls home) but anyone in cognitive science, education, or simply those that want to enhance the quality of thinking in their everyday lives. As a large body of CT research has focused on its conceptualisation and enhancement through educational strategies, this Special Issue provides a unique scope by exploring the application of CT to real-world settings and everyday life through a collection of original research, a review of the literature, and position pieces regarding topics of utmost relevance to such applications.

Specifically, in the Research section, Saiz and Rivas (2023) propose a research project focused on examining the relationship among CT, personal well-being and lifelong formation as an integrated approach to real-world problem-solving. The proposal takes a refreshingly holistic approach to CT, with the authors tackling rationale-building through not just the established cognitive, CT-focused literature but also insights from philosophy, technology development and assessment of social trends. Indeed, CT research has, arguably, always valued the concept of becoming (i.e., forming) a critical thinker, if for no other reason than personal well-being, but the manner in which this relationship is presented here—exemplified in light of real-world examples—really lands with respect to cementing the purpose of this relationship.

Guamanga et al. (2023) discuss our daily decision-making efforts through the notion of inference to the best explanation (IBE) in light of both people’s vulnerability to bias and errors of judgment. Subsequently, the authors discuss IBE’s role in the ARDESOS-DIAPROVE programme, which provides some useful insights on approaches to training CT. Particularly interesting was focused discussion on ‘explanation’ from an epistemological standpoint and how understanding the nature of explanation, through metacognitive processes such as CT, can help facilitate the development of problem-solving skills.

Hačatrjana and Namsone (2024) address the aforementioned issues of conceptualisation and training of higher-order thinking skills associated with CT in an applied manner, specifically, in the context of primary and secondary education policy. Specifically, they aim to distinguish the various thinking skills associated with CT to make them more readily accessible and approachable for students and teachers in the real-world context of everyday classroom work. As part of this, they address numerous questions, one of which I found particularly important from the perspective of engaging real-world considerations: that is, how are the concepts of thinking and reasoning as defined in policy documents reflected in curriculum descriptions across different disciplines?

Cui and Zhao’s (2024) contribution highlights the importance of not only how CT is conducted as a (meta)cognitive process, but also how that thinking is communicated, such as in real-world settings. From the standpoint of operationally defining CT, the assessment of CT performance is vital. In this context, they identify dialogue as the most commonly used means of communication and propose a qualitative coding scheme for CT in dialogue.

In the Review section, Butler’s (2024) contribution also focuses on the nature of operationalising CT through assessment. She conducts a deep dive on the current landscape of CT measures in light of strengths and weaknesses while interestingly framing it with respect to how such measures can be a useful predictor of negative life events. Coupled with real-world examples of CT’s utility, she uses this observation as an impetus for educators and educational institutions to make efforts to prioritise the *measurable* improvement of CT and, beyond the ivory tower of third-level education, for facilitation for wider populations to access CT materials and resources for their own independent learning.

Bensley (2023) performs a deep dive on the relationship between CT and intelligence with respect to real-world judgment and belief. Specifically, he highlights that, despite some conceptual overlap, CT can, perhaps, better account for trading in real-world information that we use, on a daily basis, in the formation of beliefs and judgments. That is, those who engage in CT more regularly are less likely to form unsubstantiated beliefs. To some extent, this may be a result of our common multi-dimensional view of CT, which allows for understanding and assessment beyond that of what intelligence tests typically assess—the latter, Bensley highlights, is utilised in few extant studies on unsubstantiated beliefs.

In the final section, Dumitru and Halpern (2023) provide both a very timely and interesting discussion on the applied importance of CT in light of evolving work dynamics, with particular focus on artificial intelligence and job automation. They present CT as a ‘job-proof skill’ that, once developed, makes the thinker not only a valuable asset with respect to their position in the job market but also in terms of their role in citizenship (despite reinforcing this value sometimes being a challenge). Dumitru and Halpern further recommend ways in which CT can be enhanced to help support job markets.

Eigenauer (2024) then discusses the potential of enhancing CT through specific-purpose ‘mindware’, referring to modular knowledge to be applied in appropriate contexts, akin to a heuristic. Though not novel from a cognitive psychology standpoint, its recommendation in a CT context most certainly is, as what it proposes, on the surface, seems radically contrary to what we know CT to be and endorse. However, since reading this thought-provoking article, I have personally cited it as a promising means of fighting proverbial fire with fire with respect to using CT-mindware training to combat heuristic-based, gut-level decision-making.

Next, Sternberg and Hayes (2025) utilise the former’s triangular theory of love as a basis for understanding our ‘love and hate’ for ideas and how these emotional implications can impact CT. From such discussion, they propose a model of how peripheral cognitive processes—in this context, broadly consumed within attitude and affect—can influence CT. Notably, Sternberg and Hayes make explicit the point that I think is core to the whole of this Special Issue: CT in the real world often bears little resemblance to that shown in tests or in school—if CT is to be taught, it should be performed so in reference to how it exists in the world, not in rarefied settings.

Finally, I also contribute a manuscript regarding what seemed to me to be receiving a glaring lack of attention in CT research (Dwyer 2023): whereas most of the aforementioned conceptual papers make a point of all the skills, dispositions, and practice one needs to conduct CT, seldom do I find that such manuscripts really enter into the discussion of barriers and impediments to CT. Thus, my manuscript focuses on the negative impact that some factors that we often take for granted or ignore in real-world scenarios can have on CT application—particularly, epistemological misunderstanding, too much intuitive judgment, and our ever-present bias and emotion. *That said*, I was delighted to see, in response to the call for papers to be included in this Special Issue, specifically geared towards everyday settings, so many manuscripts submitted that explicitly address barriers to CT (e.g., Bensley 2023; Guamanga et al. 2023; Sternberg and Hayes 2025, just to name a few).

Perhaps that is why, in addition to simply ‘filling a gap in research’, consideration of CT in everyday settings is so important—it highlights the use of CT in very practical settings (with all of its supports and barriers) and strips away the façade of the ‘ideal’ that is so often associated with how CT should be taught in educational settings. These ideas and their collection in the manuscripts that make up this Special Issue engage the messy world of decision-making, head-on, for what it is and offer useful advice for how to navigate this rocky terrain. With that, I hope you enjoy these articles as much as I enjoyed collating them and collaborating with their authors as part of this collection. I hope they reinforce your interest in CT as much as they have mine.
